# Challenges in assessing bone health in early infancy: a narrative review of existing technologies

**DOI:** 10.3389/fendo.2025.1651094

**Published:** 2025-10-21

**Authors:** Giorgia Pepe, Letteria Anna Morabito, Domenico Corica, Tommaso Aversa, Virginia Beretta, Elena Scarpa, Chiara Petrolini, Andrea Dall’Asta, Federica Grassi, Anna-Mariia Shulhai, Anna Maria Papini, Maria Cristina Albertini, Silvia Carloni, Serena Benedetti, Giuseppe Maglietta, Matteo Puntoni, Caterina Caminiti, Tullio Ghi, Maria Elisabeth Street, Serafina Perrone, Malgorzata Wasniewska

**Affiliations:** ^1^ Department of Human Pathology of Adulthood and Childhood, University of Messina, Messina, Italy; ^2^ Neonatology Unit, Department of Medicine and Surgery, Pietro Barilla Children’s Hospital, University of Parma, Parma, Italy; ^3^ Department of Surgical Sciences, Obstetrics and Gynecology Unit, University of Parma, Parma, Italy; ^4^ Department of Medicine and Surgery, University of Parma, Parma, Italy; ^5^ Department of Chemistry “Ugo Schiff”, University of Florence, Sesto Fiorentino, Italy; ^6^ Department of Biomolecular Sciences, University of Urbino Carlo Bo, Urbino, Italy; ^7^ Clinical and Epidemiological Research Unit, University Hospital of Parma, Parma, Italy; ^8^ Unit of Paediatrics, University Hospital of Parma, Parma, Italy

**Keywords:** bone mineral density, early infancy, REMS technology, ultrasound, DXA

## Abstract

To date, no shared guidelines have been approved for the diagnosis and management of low bone mineral density (BMD), especially in early infancy. Therefore, there is an increasing demand for new methodologies to allow the assessment of bone health status in this specific cohort, which is exposed to several risk factors (e.g. maternal vitamin D deficiency, pregnancy-associated diseases, preterm birth and comorbidities, low birth weight, intrauterine growth restriction). Currently, the assessment of BMD in newborn and infants relies mainly on serum and urinary biochemical markers, in association with several technologies to measure bone mineral content, such as dual-energy X-ray absorptiometry (DXA) and quantitative ultrasound (QUS) being traditionally used, despite many limitations. More recently, Radiofrequency Echographic Multi-Spectrometry (REMS) emerged as a promising tool in clinical practice for screening and monitoring BMD. Due to the radiation-free technology, an extremely ease of use, low costs, an excellent degree of sensitivity, specificity, and reproducibility, REMS technology has proven to be the gold standard technique in sensitive populations such as pregnant women, newborns and infants, allowing mass extended screening strategies. However, to date no validate cut-off reference for REMS in paediatric age are available. Future longitudinal studies on REMS methodology are needed to build reference standards and new shared algorithms, combining biochemical and instrumental data, for the diagnosis, management and treatment of decreased BMD before and after birth.

## 1 Introduction

**Figure 1:**
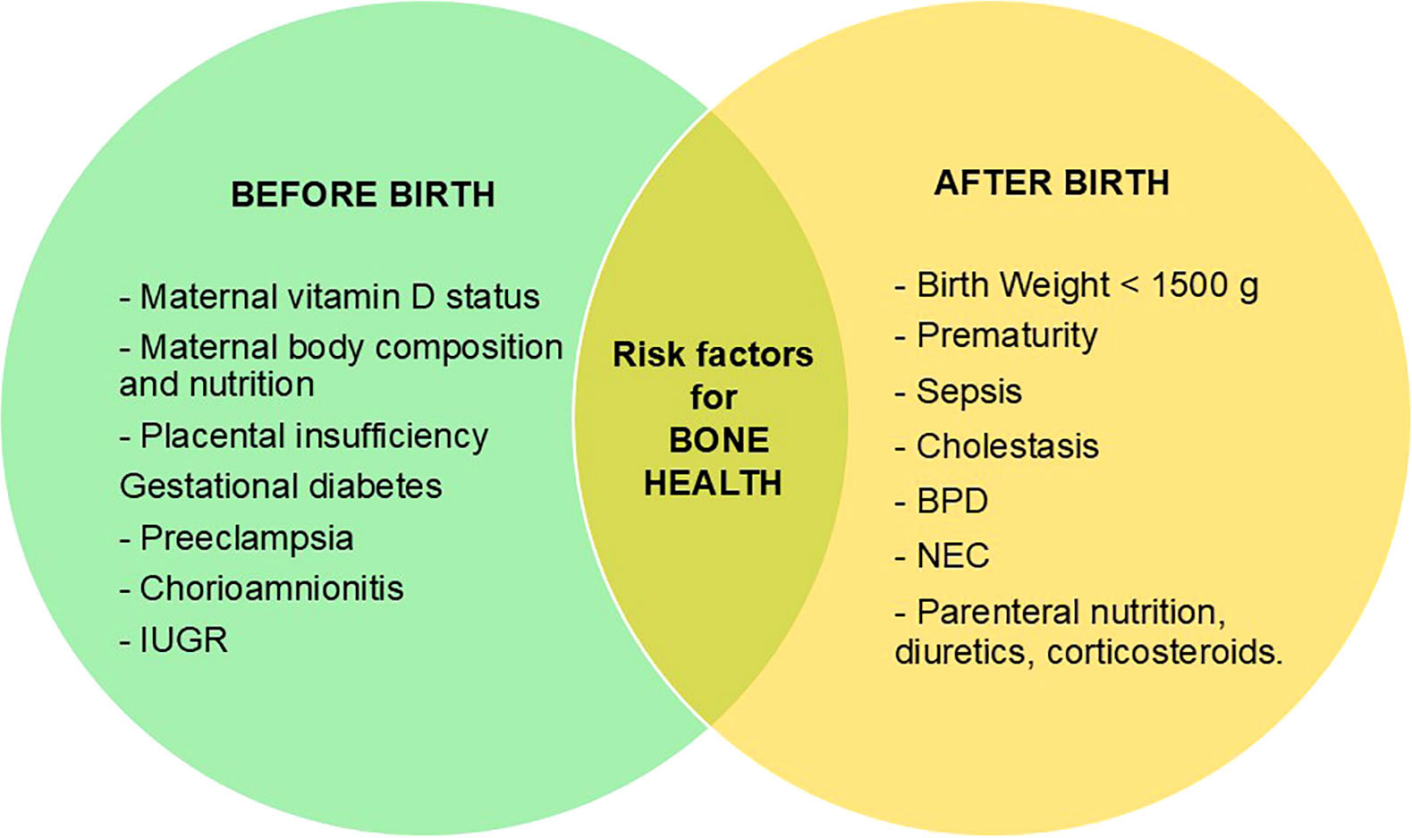
Factors influencing negatively bone health status before and after birth. IUGR, intrauterine growth restriction; BPD, Bronchopulmonary dysplasia; NEC, Necrotizing enterocolitis.

The term “bone health” usually refers to bone’s strength, expressed as fracture resistance and measured by bone mineral reserve (1, 2). However, bone health appears to be more complex, including also all the intrinsic and extrinsic factors that may contribute to it, already during prenatal life. As a result, bone health relies on a sophisticated interplay between biological, genetics, metabolic, hormonal, environmental, mechanical, and nutritional factors, whose intricate interaction starts in the early stages of intrauterine life, continuing throughout childhood, and peaking between the second and third decade of life, when “peak bone mass” is reached (3). Indeed, it is well known that the main factors determining bone health exert their influence since prenatal age and mainly during the third trimester of pregnancy (**Figure 1**), when up to 80% of fetal bone mineral accumulation occurs, and a progressive expansion of bone volume takes place through an increase in trabecular thickness and cortical architecture under the control of mineral availability (4). Therefore, most of the information on factors affecting bone health in early childhood comes from studies carried on premature infants. Since the great majority of bone mineralization occurs during the third trimester, preterm birth represents per se a risk factor for decreased bone mass: the sudden interruption of transplacental transport of calcium and phosphate due to premature birth and the co-occurrence of conditions such as sepsis, necrotizing enterocolitis, cholestasis, bronchopulmonary dysplasia, etc., as well as low birth weight (less than 1500 g) or intrauterine growth restriction (IUGR) make premature babies more at risk of developing reduced bone mineralization in later life (5).

In recent years, expanding knowledge of fetal bone development has led to the identification of a growing number of endocrine and non-endocrine factors that play a key role in ensuring bone health.

The role of leptin, cytokines, oxidative stress (OS) and endocrine disrupting chemicals (EDCs) on bone formation and resorption is emerging, in addition to the pregnancy specific regulation of calcium-phoshate metabolism and the well-known influence of cortisol, GH/IGF-1 axis and vitamin D status. In particular, cytokines (IL-1, IL-6 and TNF-α) and oxidative stress exert their negative effects by impairing osteoblast differentiation and bone remodeling in favor of resorption. Likewise, high plasma levels of EDCs, such as poly- and perfluoroalkyl substances (PFAS), in first 1000 days of life are associated with lower bone mineral density (BMD) SDS at age of 3 years (5–8). Furthermore, these substances seem to have long-term effects on bone health through multiple epigenetic mechanisms and gene expression modulation. In addition, maternal issues during pregnancy, such as vitamin D deficiency, impaired body composition and nutrition, pregnancy-related diseases, have been widely reported as influencing fetal bone mass and BMD and peak bone mass in adulthood, even though the pathogenic mechanisms of fetal endocrine programming is not yet completely understood (9–13).

It is therefore quite necessary to implement effective strategies for bone health prevention, starting with the identification of the categories of patients most at risk, including the application of new technologies that could be non-invasive, easily, and longitudinally performed, suitable and applicable in early childhood, to assess any changes in bone health status (14, 15). Over the years, several technologies have been proposed to measure bone mineral content, including dual-energy X-ray absorptiometry (DXA) and quantitative ultrasound (QUS), which are traditionally used (16–20). Most recently, Radiofrequency Echographic Multi-Spectrometry (REMS) has emerged as a promising tool in clinical practice for screening and monitoring BMD.

Aim of this narrative review is to summarize the state of the art on technologies currently available for the assessment of bone health in the early infancy, focusing on new emergent methodologies for early identification, stratification, and management of osteopenia in this specific cohort of patients.

## 2 The main technologies to assess bone health in early infancy

### 2.1 Dual x-ray absorptiometry and x-ray

Dual X- ray absorptiometry (DXA) is a speed, precise, safety, relatively low-cost technique and it has been considered recently as the gold standard for the evaluation of bone density parameters.Since its introduction in clinical practice, DXA scans have been performed both in infants and children, and numerous research studies validated its precision and accuracy (16, 21, 22).

Two DXA parameters, BMC (bone mineral content) and BMD, provide informations on the state of bone health through the analysis of different X-ray absorption by the bone, subtracting soft tissue components.

In clinical practice, the reference parameter is Z-score, defining the number of standard deviations of the patient’s bone density with respect to a reference population of the same age, gender and ethnicity.

Due to the rapid growth characterizing the early age of life, the information about BMD in children under 3 years of age is mainly obtained through the evaluation of whole-body measurements, while the posterior anterior lumbar spine scans are less frequently used under 5 years of age. Although both the sites have been validated by current recommendations of the International Society for Clinical Densitometry (ISCD) (23), areal BMD measurement should not be used routinely in infants (difficulty to place the babies in appropriate positioning, scarce uniformity of bones in the three dimensions secondary to the rapid growth process) (24).

There are some important limitations to the use of DXA in early infancy: in addition to limited availability secondary to cumulative radiation exposure, the accuracy of DXA is also affected by technical and operator variability, with significant variation in the parameters reported for a subject due to different skills and software used for analysis. In addition, variations in height, skeletal size and shape and the amount of soft tissue that occurs during the rapid growth of infants may limit the comparative evaluation of DXA scans at various ages. In children, DXA BMD measurements are influenced by height, so bone mineral apparent density, and height-for-age Z score are used and recommended to reduce the confounding effect of short stature on spine bone density (16, 25–27).

Overall, X-rays have limited application in assessing bone status. According to the literature, X-rays could be used to identify significant signs of osteoporosis or bone fractures, but they are not suitable for early diagnosis: some forms of osteoporosis with bone loss <20-40% may not be evident with this technique, and significant degrees of demineralization or fractures may be absent at an early stage (28). Despite this, the Koo score (29) is still used to describe the radiological features of metabolic bone disease (MBD) in premature infants.

### 2.2 Quantitative ultrasound

Quantitative Ultra Sound (QUS) is an non-invasive, unexpensive, portable and radiation-free method to assess bone density in children, especially for very young pediatric populations, where the use of traditional techniques, such as DXA may be less appropriate considering the exposition to ionizing radiation (30). It assesses both BMC and quantitative properties of bone (cortical thickness, microarchitecture, and elasticity), providing comprehensive information on “bone strength” through the evaluation of two parameters: speed of sound (SOS) and bone transmission time (BTT), depending on the velocity or attenuation of the ultrasound waves through the bone tissue (29, 31). QUS could be used in the assessment of bone mineral status in both preterm infants and children and appropriately in the evaluation of MBD. Most QUS devices are designed to be positioned only on a single skeletal site (e.g. proximal phalanges of the hand, heel, radius and/or tibia), but a multisite QUS device is also available, with different probes on one or more skeletal sites, which in children are usually the tibia (midshaft) and radius (distal third) (20, 32–34).

Althoug QUS devices are suitable for pediatric patients, there are currently insufficient data to determine whether this technique is equivalent to DXA in providing an estimate of bone health, and the limited information available from comparing BMD measured with QUS and DXA has shown conflicting results.

Furthermore, the absence of reliable reference values for pediatric age, the impossibility to using it for the axial skeleton, and technological diversity among QUS devices, both in terms of measurements sites and bone parameters, represent a major problem in the widespread use of QUS in clinical practice (35–38).

### 2.3 Radiofrequency echographic multi spectrometry

The most recently validated instrumental technique to measure bone mineral status is known as Radiofrequency Echographic Multi-Spectrometry (REMS). REMS is a non-invasive radiation-free methodology, based on the use of ultrasound (39–41).

In adults, REMS technology enables the assessment of axial BMD by a rapid ultrasound scan of lumbar vertebrae (80 s scan) and femoral neck (40 s scan), which represents central anatomical reference sites (42).

The basic principles of this technology consist in a combination between radiofrequencies signals and ultrasound imaging, acquired by a transducer. Simultaneous acquisition of radiofrequencies (native unfiltered ultrasound signals) allows to obtain all available information about the site studied, resulting in more precise and complete acquisition than other conventional ultrasound-based approaches.

The unfiltered radiofrequency signals acquired are then processed by a fully automatic algorithm, transformed into a specific spectrum of the patient, and compared with previously established reference spectral models matched by gender, age and BMI of healthy and osteoporotic bones (39, 43).

Starting from a simple and fast ultrasound scan, this approach allows to obtain quantitatively and qualitatively relevant information about bone health status. Indeed, in addition to quantitative parameters provided by DXA examination, REMS technology provides also a measure of bone quality through the Fragility score, a system validated to estimate 5-years prediction of fracture risk (44).

The 5-year follow-up study by Pisani et al. showed that REMS fragility score to be superior to the only BMD in fracture risk prediction for femur and spine, thanks to the additional information conveyed by REMS technology (45).

Di Paola et al. (46) compared REMS methodology with DXA for osteoporosis diagnosis, enhancing a satisfactory accuracy and precision. Interestingly, the high level of precision of REMS indicates a low intra-operator variability, which represents one of the main advantages of this technology.

Likewise, the REMS methodology showed a specificity and sensitivity (90.4% and 95.5%, respectively) comparable to DXA at femoral neck evaluation. Furthermore, studies in both Caucasian and Japanese subjects recently enhanced a potentially more accurate measure of REMS BMD versus DXA BMD, thanks to the possibility to automatically ignore artefacts due to calcifications, osteophytes, fractures, etc. (47–49).

Notably, the non-ionizing radiation technology and the high rate of reproducibility of REMS examinations makes this technique suitable for regular monitoring of BMD, both in primary prevention and in tracking therapeutic responses. Moreover, the extremely ease of use, the portability of the device, and the lower costs allow REMS methodology to be successfully employed in several healthcare settings, minimizing operator-dependent bias (41).

Accordingly, the several advantages of REMS support the use of this technology as a valuable alternative to DXA and QUS in bone health evaluation, especially in sensitive populations, such as the foetus and the newborn, which enable to safely fulfill extended mass screening strategies. However, the use of REMS is only partially known and shared in clinical practice to date, especially in early infancy. De Gennaro et al. validated REMS methodology in pregnant women, suggesting REMS as the new gold standard for the evaluation of the BMD in this specific cohort (37, 40, 50).

Data on the use of REMS in the newborns are extremely scarce and consequently reference models and population-based data are still lacking. In this regard, Perrone et al. proposed an algorithm which emphasizes the use of REMS during prenatal and postnatal life, in presence of maternal and fetal risk factors. This model is based on the association of echographic data with serum and urinary markers of bone metabolism to determine bone mineral status (51). Very recently, the same authors developed a pioneering study protocol to evaluate and standardize REMS BMD from intrauterine to extrauterine life. It consists in a multicenter clinical trial - currently ongoing- and included 200 mother-newborn dyads, with REMS follow-up planned until 12 months of age (52). Of course, to get an accurate and precise measurement in newborn and infants, it could be advisable to hold the baby still during the scanning by using immobilisation devices, parents, and/or staff, and to make repeated scans of the same site. Indeed, due to its safe and easy use, REMS technology could contribute to improve the knowledge of bone health before and after birth, thus allowing effective prevention strategies and stratification of the risk of fractures, with valuable insights for both obstetric and neonatal care (e.g type of delivery, type of intervention for the shoulder dystocia, specific programs for newborns with low bone mineral status).

## 3 Discussion

The accumulation of “bone mass”, which is a determining factor in bone strength and fracture risk, takes place during a delicate “time window” that begins during intrauterine life, and extends from childhood to early adulthood, representing an important period for achieving maximum growth and development of bone mineral tissue. Currently, bone health assessment cannot be separated from the analysis of serum and urinary biochemical markers, whose levels are reliable indicators of bone health and turnover, useful in identifying conditions associated with decreased BMD. However, there are still significant limitations for early diagnosis of MBD, even in at-risk categories.

In recent years, most research has focused on identifying screening strategies to measure bone mineral status in targeted populations, known to be more exposed to risk factors for osteopenia, such as premature birth, low or very low birth weight, IUGR, comorbidities of prematurity, total parenteral nutrition, maternal vitamin D deficiency, and several pregnancy-associated diseases (e.g., gestational diabetes). However, to date there are no shared guidelines or universal consensus for the diagnosis and management of MBD, particularly in early childhood.

In addition to already known pathological conditions, there are other factors that appear to influence bone health and strength, thus modulating lifetime risk of osteoporosis, such as the recently discovered epigenetic effects of fetal programming, OS and EDCs, also in full term healthy babies (51, 53–55).

The measurement of BMD in early infants is a controversial issue, due to the limitation of current diagnostic techniques in detecting early markers and/or signs of MBD, which is usually diagnosed in advanced stages, when there is a consistent lack of the expected mineralization for age. To date there is no universally accepted method for a large-scale screening of bone health, mainly because most techniques used for BMD measurement require ionizing radiation, instrumental dimensions are often inadequate for infants, and the time required to motion artifacts represent an unresolved issue. Nevertheless, there is an urgent need for non-invasive screening programs for the implementation of prevention strategies and early identification of BMD alterations (11–14, 51, 56, 57).

Currently, DXA and QUS are traditionally used, despite several limitations (**Table 1**). Although DXA remains the gold standard technique for evaluating bone health, the issue of radiation exposure and the rapid changes in skeletal size may limit its application in early age. The use of QUS has been implemented in recent years, probably due to its accessibility and safety, but its validity in measuring BMD in early childhood is still a matter of debate. The lack of universal QUS threshold values and validated reference cut-offs, the differences in ROIs and bone properties measured, and high percentage of classification errors compared to DXA scans make these techniques non-interchangeable in assessing the bone status of children (36, 58–60).

**Table 1:** Key points of the main technologies to assess bone health in early infancy.

Technologies	Advantages	Disadvantages
DXA	Gold standard, widely used. Cut-off values available for pediatric age. Measures bone quantity (BMC and BMD). Good precision and accuracy. Medium cost.	Ionizing radiation exposure. Not portable. No information on bone quality. Intra-operator variability.
QUS	Radiation-free. Measures bone quantity by computing SOS and BTT. Easy to use. Portable. Low cost.	Single site (usually) - Not suitable for axial skeleton. Not well-defined accuracy in BMD estimation in pediatric age. Lack of reference cut off for pediatric age.
REMS	Radiation-free. Measures bone quantity and quality (BMD, fragility score). High precision and accuracy. Easy to use. Portable. Low cost.	Not whole body measurement. Lack of reference cut off for pediatric age. Not widely used and shared in clinical practice.

DXA, Dual X- ray absorptiometry; QUS, Quantitative Ultra Sound; REMS, Radiofrequency Echographic Multi-Spectrometry; BMC, bone mineral content; BMD, bone mineral density; SOS, speed of sound; BTT, bone transmission time.

More recently, REMS has been proposed as an innovative ultrasound-based technology with valuable insights in several clinical settings. Over the last years, studies carried out in adulthood underlined that REMS is a promising and ductile methodology, relying on a specificity and sensitivity highly comparable to DEXA at femoral neck evaluation, together with a satisfactory degree of accuracy and precision (42, 46, 47). Moreover, when compared to other densitometric techniques, REMS technology showed a potential superiority, providing not only traditional quantitative parameters (BMD, T-score and Z-score), but also qualitative estimation of bone quality through the Fragility score (44, 45). These features, in addition with a high rate of reproducibility, make REMS BMD measurements suitable for short-term therapeutic monitoring, overcoming the temporal limits existing for other densitometric techniques, which typically require a minimum interval of at least 1 year between two scans. Due to the non-ionizing radiation methodology, an extremely ease of use, a high rate of reproducibility, and low costs, REMS appears to be the elective technology for BMD screening, even in sensitive populations such as in pregnancy and childhood.

Despite these advantages, there are some limitations in the application of REMS methodology on a large scale of patients, including the impossibility to obtain “whole body” measurements, which could be useful in early childhood because of the rapid growth of body. Above all, reference limit values for measurements of BMD with REMS in early childhood is still missing. The lack of information about the distribution of BMD in newborns limits the application of this methodology, but preliminary and encouraging data from ongoing research studies may support the validity of BMD Z-score measurement in a single site to evaluate bone status (52).

In the future, the integration of REMS in early MBD screening could have important insights, taking into account the actual absence of a technique giving complete information on bone mineral quality in newborns and infants. Of course, the identification of early physiological and non-physiological variations of bone structure through REMS may have long-term implications for lifelong skeletal health, thus offering unique information not always accessible via traditionally used imaging techniques.

In conclusion, innovative, non-invasive and ductile technologies, such as REMS methodology, would open new scenarios to significantly improve neonatal/pediatric care with screening strategies for bone health assessment, resulting in a potential reduction in MBD. and risk of long-term fractures.

Future longitudinal studies on this issue are needed to allow the building of new shared algorithms and dedicated software, combining biochemical and instrumental data, for the diagnosis, management and treatment of decreased BMD in early infancy.
